# The VP1u Receptor Restricts Parvovirus B19 Uptake to Permissive Erythroid Cells

**DOI:** 10.3390/v8100265

**Published:** 2016-09-28

**Authors:** Remo Leisi, Marcus Von Nordheim, Carlos Ros, Christoph Kempf

**Affiliations:** 1 Department of Chemistry and Biochemistry, University of Bern, Bern 3012, Switzerland; marcus.vonnordheim@dcb.unibe.ch (M.V.N.); carlos.ros@dcb.unibe.ch (C.R.); christoph.kempf@dcb.unibe.ch (C.K.); 2 CSL Behring AG, Bern 3014, Switzerland

**Keywords:** parvovirus B19, virus entry, VP1u, VP1u receptor, tropism, erythropoiesis, virus mimicry, MS2 bioconjugation

## Abstract

Parvovirus B19 (B19V) is a small non-enveloped virus and known as the causative agent for the mild childhood disease *erythema infectiosum*. B19V has an extraordinary narrow tissue tropism, showing only productive infection in erythroid precursor cells in the bone marrow. We recently found that the viral protein 1 unique region (VP1u) contains an N-terminal receptor-binding domain (RBD), which mediates the uptake of the virus into cells of the erythroid lineage. To further investigate the role of the RBD in connection with a B19V-unrelated capsid, we chemically coupled the VP1u of B19V to the bacteriophage MS2 capsid and tested the internalization capacity of the bioconjugate on permissive cells. In comparison, we studied the cellular uptake and infection of B19V along the erythroid differentiation. The results showed that the MS2-VP1u bioconjugate mimicked the specific internalization of the native B19V into erythroid precursor cells, which further coincides with the restricted infection profile. The successful mimicry of B19V uptake demonstrates that the RBD in the VP1u is sufficient for the endocytosis of the viral capsid. Furthermore, the recombinant VP1u competed with B19V uptake into permissive cells, thus excluding a significant alternative uptake mechanism by other receptors. Strikingly, the VP1u receptor appeared to be expressed only on erythropoietin-dependent erythroid differentiation stages that also provide the necessary intracellular factors for a productive infection. Taken together, these findings suggest that the VP1u binds to a yet-unknown erythroid-specific cellular receptor and thus restricts the virus entry to permissive cells.

## 1. Introduction

Parvovirus B19 (B19V) is a human pathogen with a worldwide prevalence. The transmission of the virus occurs via the respiratory route and the infection generally leads to mild courses as the childhood disease *erythema infectiosum* [1]. However, B19V infections in immunocompromised people or individuals with hemolytic disorders may become chronic and cause severe manifestations. The cellular tropism of B19V is strongly restricted to intermediate erythroid differentiation stages, where the susceptibility increases from erythroid progenitors to erythroblasts [2]. Studies using ex vivo expanded cluster of differentiation (CD) 34+ hematopoietic stem cells show the highest permissivity in erythropoietin-dependent erythroid differentiation stages—colony forming unit-erythroid (CFU-E), proerythroblasts and early basophilic erythroblasts [3,4,5,6]. The extraordinarily narrow tropism is supposed to be determined by both a restricted receptor expression and intracellular factors [7].

The current state of the literature suggests that B19V requires multiple receptors to achieve the uptake of the virus into the erythroid host cells. The glycosphingolipid globoside, also known as P antigen, was found as a cellular receptor for B19V [8]. Globoside was shown to be essential for in vitro as well as in vivo infections [9], but is not sufficient to trigger the cellular uptake of the virus [5,10]. Therefore, it is believed that globoside serves as a primary receptor that mediates the initial attachment of B19V on the target cells and further facilitates tissue spreading of the virus [11,12]. In addition to globoside, the virus needs the binding to a specific entry receptor that triggers the endocytosis of the capsid. The membrane proteins α5β1 integrin [13] and Ku80 [14] have been proposed as possible coreceptors, however, their expression patterns only vaguely correlate with the B19V tropism and cannot explain the restricted internalization profile of the virus [5,15,16].

B19V is a small, non-enveloped virus with a single stranded DNA genome. The capsid is composed of 60 almost identical viral proteins, forming an icosahedral symmetry. About 57 of the 60 protomers are viral protein 2 (VP2), also referred to as major capsid protein [17,18]. The remaining three VP1 proteins contain the common VP2 region plus an additional N-terminal extension of 227 amino acids (aa), called the VP1 unique region (VP1u). The VP1u represents an immunodominant part of the virus and contains several clusters of epitopes which become the target of neutralizing antibodies during the infection [19,20,21,22].

Recently, we reported that the VP1u region of B19V selectively binds and internalizes into erythroid cell lines [15]. Using the VP1u as a targeting module, we specifically delivered fluorophores, DNA and toxins to erythroid precursor and erythroleukemic cells, demonstrating a possible diagnostic and therapeutic application of this viral protein [16]. Next, we localized the receptor-binding domain (RBD) in the N-terminal part of the VP1u (aa 5–80), which correlated with the profile of the found neutralizing epitopes in this region [23]. Finally, we identified a rigid α-helical fold in the RBD and found a putative and conserved receptor binding site between helix 1 and helix 3. Taken together, the results suggested that B19V uses the RBD in the VP1u to interact with a yet unknown erythroid-specific entry receptor, which triggers the endocytosis of the virus.

To investigate whether the VP1u is sufficient to internalize a viral structure, we sought to mimic B19V with an unrelated capsid. To this end, we chose the bacteriophage MS2 capsid as structural core since MS2 (i) has a similar size as B19V; (ii) does not bind to eukaryotic cells; (iii) is easily produced in *Escherichia coli* with high yields and purity [24]; and (iv) was already used for similar approaches as a drug or gene delivery vehicle [25,26]. The results with the MS2-VP1u bioconjugate showed that the VP1u is sufficient to internalize a virus capsid into host cells. Furthermore, we resolved the B19V internalization profile along the erythroid differentiation and found a direct correlation between virus uptake and VP1u receptor expression on permissive cells.

## 2. Materials and Methods

### 2.1. Cells

The megakaryocyte-erythroid UT7/Epo cell line was kindly provided by Eiji Morita (Tohoku University School of Medicine, Sendai, Japan) and cultured, in RPMI 1640 media (Thermo Fisher Scientific, Waltham, MA, USA), 5% fetal calf serum (FCS) (Bioconcept, Allschwil, Switzerland), 2 U/mL recombinant human erythropoietin (rhEPO) (Janssen-Cilag, Zug, Switzerland). HeLa, MRC-5, K562 and CD34+ hematopoietic cells isolated from bone marrow were all obtained from the American Type Culture Collection (ATCC). HeLa and MRC-5 cells were cultured in Dulbecco’s modified Eagle’s medium F-12 (DMEM/F-12) (Sigma-Aldrich, St. Louis, MO, USA), 5% FCS; K562 cells were cultured in Iscove’s modified Dulbecco’s medium (IMDM) (Sigma-Aldrich), 10% FCS. All cell culture media were supplemented with l-alanyl-l-glutamine (Biochrom, Berlin, Germany) and penicillin–streptomycin (Biochrom). For erythroid differentiation, CD34+ bone marrow cells were expanded in alpha minimum essential media (αMEM) (Thermo Fisher Scientific) with 20% BIT 9500 (BSA, insulin, transferrin), 90 ng/mL ferric nitrate (Sigma-Aldrich), 900 ng/mL ferrous sulfate (Sigma-Aldrich), 100 ng/mL stem cell factor (SCF) (Thermo Fisher Scientific), 5 ng/mL interleukin (IL)-3 (Thermo Fisher Scientific), 1 µM hydrocortisone (Sigma-Aldrich), and 3 U/mL rhEPO. For stem cell expansion, CD34+ cells were cultured for seven days in αMEM, 20% BIT 9500, supplemented with StemSpan CD34+ Expansion Supplement (10X) (StemCell, Vancouver, BC, Canada), containing the human growth factors FMS-like tyrosine kinase 3 ligand (Flt3L), SCF, IL-3, IL-6, and thrombopoietin (TPO). All cells were cultured at 37 °C and 5% CO_2_. Peripheral blood was obtained from a blood transfusion service of the Swiss Red Cross (BSD SRK, Bern, Switzerland).

### 2.2. Parvovirus B19-Infected Plasma

Human plasma from a parvovirus B19 infected person (5 × 10^9^ virions/µL, genotype 1) was obtained from our donation center (CSL Behring AG, Bern, Switzerland).

### 2.3. VP1u Expression

The recombinant VP1u was expressed and purified as previously described [15,16]. The VP1u sequence, derived from the infectious clone pB19-M20 (Susan Wong, National Institutes of Health, Bethesda, MD, USA), was cloned into the pT7-FLAG-MAT-Tag-2 expression vector (Sigma-Aldrich). Plasmid-transformed BL21(DE3) *E. coli* were grown in lysogeny broth media containing ampicillin until the optical density measured at a wavelength of 600 nm (OD_600_) reached an absorbance of ~0.5. VP1u expression was induced with 1 mM isopropyl-β-d-thiogalactopyranoside (IPTG) (Sigma-Aldrich) and carried out for 4 h at 37 °C. Collected cell pellets were resuspended in phosphate buffered saline (PBS) containing 10 mM imidazole and sonicated for 15 × 10 s on ice. The lysate was cleared by centrifugation (10,000× *g*, 4 °C) and soluble recombinant VP1u was purified twice with nickel nitrilotriacetic acid (Ni-NTA) agarose as previously described [15,16]. The recombinant VP1u (1.5 mg/mL) was eluted in PBS with 250 mM imidazole, reduced with 5 mM tris(2-carboxyethyl)phosphine (TCEP) and dialyzed twice with 7 kDa MWCO Zeba columns (Thermo Fisher Scientific) against PBS pH 6.8.

### 2.4. MS2 Capsid Expression

The MS2 coat protein sequence was obtained from GenScript (Nanjing, China) and transformed as plasmid into *E. coli* BL21(DE3). MS2 protein expression was initiated at an OD_600_ of ~0.5 and proceeded overnight. After sonication (15 × 10 s) on ice and clearance of the lysate (10,000× *g*, 4 °C), MS2 capsids were purified twice by ultracentrifugation through 20% sucrose (100,000× *g*, Beckman Coulter, Optima MAX-XP; TLA 100.3 rotor, 1 h, 4 °C). Capsids were resuspended in PBS and dialyzed twice using 40 kDa MWCO Zeba columns equilibrated with PBS pH 7.4. Purity of the capsids was checked by agarose gel electrophoresis (AGE) and sodium dodecyl sulfate polyacrylamide gel electrophoresis (SDS-PAGE). Concentration of the protein:RNA complexes was determined by NanoDrop (NanoDrop2000, Thermo Fisher Scientific). The absorption of the MS2 suspension A260:A280 (1.66) indicated a protein:RNA ratio of about 65%:35%. Based on this ratio the MS2 protein concentration was adjusted to 10 mg/mL.

### 2.5. MS2-VP1u Bioconjugation

The lysine reactive fluorescent dyes (NHS-Atto488; NHS-Atto633) were dissolved in dimethyl sulfoxide (DMSO) and added each at a 40-fold molecular excess to the purified MS2 capsids (10 mg/mL). The coupling reaction was carried out for 2 h at room temperature in PBS (pH 7.4). Non-reacted dye was removed by two passages through 40 kDa MWCO Zeba columns, equilibrated with PBS pH 6.8. The bifunctional crosslinker maleimide-PEG_24_-N-hydroxysuccinimide ester (NHS-PEG_24_-maleimide; Thermo Scientific) was dissolved in DMSO and added to the Atto-labeled MS2 capsids at a 500-fold excess per capsid. Crosslinking of capsid surface lysines with the NHS-ester group was allowed for 1 h at room temperature in PBS (pH 6.8). Non-coupled crosslinker was removed by passing the mixture through 40 kDa MWCO Zeba columns thrice. Reduced VP1u was then added as a 50-fold excess to maleimide-activated MS2 capsids and incubated for 6 h at room temperature. To induce hydrolysis and quenching of the reacted or remaining maleimide groups, the suspension was adjusted to pH 8 with Tris buffer (50 mM final concentration) and incubated overnight at 4 °C. MS2-VP1u capsids were separated from non-reacted VP1u by centrifugation through 20% sucrose. All coupling reactions were carried out with protein concentrations higher than 1 mg/mL to achieve efficient crosslinking. MS2-VP1u bioconjugation was analyzed by Western blot and detected with an anti-FLAG antibody (rat; Agilent Technologies, Santa Clara, CA, USA) and secondary horseradish peroxidase (HRP)-labeled anti-rat antibody (goat; Agilent Technologies). To determine the percentage of the VP1u-crosslinked MS2 coat proteins, we analyzed the band intensities on the SDS-PAGE with the ImageJ software [27] and calculated the molar proportion of the bioconjugates per total MS2 molecules. The result suggests that ~15% of the MS2 coat proteins were conjugated with the VP1u, corresponding to about 20–30 VP1u per MS2 capsid. Bioconjugated MS2-VP1u capsids could be stored for several weeks at 4 °C without significant loss of function.

### 2.6. MS2 Internalization

Cultured or ex vivo expanded cells were harvested and washed once with PBS. For each sample, 2 × 10^5^ cells were resuspended in 50 µL PBS and incubated with 250 ng Atto-labeled MS2-VP1u (6 × 10^10^ particles) for 1 h at 37 °C. Cells were washed once with PBS, fixed with formaldehyde (2%), permeabilized with ethanol and analyzed by fluorescence microscopy (Axiovert 35, Carl Zeiss, Jena, Germany) or flow cytometry (BD LSR II, BD Biosciences, San Jose, CA, USA). Fluorescence images were processed with ImageJ software [27]; flow cytometry data was analyzed using the FlowJo software (FlowJo, Ashland, OR, USA).

### 2.7. B19V Uptake and Competition

For internalization assays with cell lines, 3 × 10^5^ cells were resuspended in 300 µL PBS and incubated with 10^11^ B19 virions from human plasma. For internalization assays along the erythroid differentiation, 2 × 10^5^ erythroid progenitor/precursor cells from each day were resuspended in 50 µL PBS and incubated with 1.5 × 10^10^ virions. After incubation at 37 °C for 1 h, cells were washed once with PBS, trypsinized for 5 min at 37 °C and washed twice again. Intracellular B19V genomes were extracted (DNeasy Blood and Tissue Kit; Qiagen, Hilden, Germany) and quantitated by iTaq SybrGreen quantitative polymerase chain reaction (qPCR) (BioRad, Hercules, CA, USA; forward primer: 5′-GGGCAGCCATTTTAAGTGTTT-3′; reverse primer: 5′-CCAGGAAAAAGCAGCCCAG-3′).

For B19V internalization competition assays, cells were resuspended in 50 µL PBS and incubated with the competitor (MS2-VP1u or VP1u) for 30 min at 4 °C prior to the incubation with B19V at 37 °C for 30 min. Afterwards, samples were processed and quantitated as described for the normal internalization assays. Efficient inhibition of B19V uptake was achieved by preincubation with equimolar concentrations of MS2-VP1u or a 100-fold excess of recombinant VP1u.

### 2.8. B19V Infectivity Assays

Ex vivo expanded erythroid precursors (10^5^ cells per sample) were resuspended in 50 µL PBS and incubated with 10^9^ virions for 1 h at 37 °C to allow virus internalization. Cells were washed once and then resuspended in the culture media they were grown and incubated for 48 h at 37 °C, 5% CO_2_. Total B19V DNA was extracted and quantitated with qPCR. For competition with recombinant VP1u, cells were preincubated for 30 min at 4 °C with 10^12^ recombinant ∆C126 or ∆N30/∆C126 VP1u molecules (1000-fold excess versus B19 virions). Since the competing VP1u was washed away after primary internalization, VP1u-competed infections were incubated only for 24 h at 37 °C to avoid secondary infections in absence of the uptake competitor.

### 2.9. Statistical Analysis

Data is shown as the mean ± standard deviation. Replicates represent values from three independent experiments.

## 3. Results

### 3.1. Bioconjugation of the MS2-VP1u Capsid

**Figure 1:**
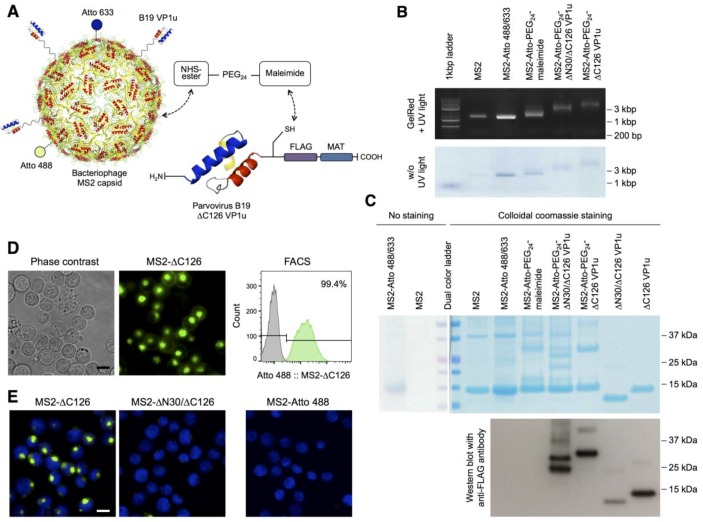
Bioconjugation of the B19 viral protein 1 unique region (VP1u) with the bacteriophage MS2 capsid, and internalization of the bioconjugate on erythroleukemic UT7/Epo cells: (**A**) Schematic depiction of the bioconjugation and labeling strategy of the bacteriophage MS2 capsid (PDB ID: 1MST [28]). The recombinant ∆C126 VP1u protein with a C-terminal metal affinity tag (MAT), FLAG tag, and a unique cysteine was coupled to the MS2 capsid using the heterobifunctional maleimide-PEG_24_-N-hydroxysuccinimide ester (NHS-PEG_24_-maleimide) crosslinker. The NHS-ester was conjugated to the surface lysines of the MS2 capsid and the maleimide group reacted with the unique cysteine in the VP1u. In addition, the capsid was labeled with the NHS-Atto 488 (green) and NHS-Atto 633 (blue) dyes; (**B**) Migration of the wild-type (WT) and bioconjugated MS2 capsids by agarose gel electrophoresis and detection of encapsidated RNA with GelRed. Capsid proteins are labeled with NHS-Atto dyes (green-blue); (**C**) Sodium dodecyl sulfate polyacrylamide gel electrophoresis (SDS-PAGE) and Western blot analysis of the MS2 capsids, recombinant VP1u proteins, and the bioconjugates. Recombinant VP1u and MS2-VP1u conjugates were detected with an anti-FLAG antibody. In the Western blot, the transfer of low molecular weights proteins was less efficient; therefore, the detected signal did not quantitatively reflect the original and relative input of the proteins; (**D**) Uptake of the fluorescent MS2-∆C126 VP1u into UT7/Epo cells and detection of the internalized capsids in living cells by fluorescence microscopy and fluorescence-activated cell sorting (FACS); (**E**) Fluorescence microscopy images of the MS2-∆C126 VP1u and the Atto-labeled but internalization-deficient controls MS2-∆N30/∆C126 VP1u and MS2 (63× magnification). Scale bar: 15 µm.

Our previous findings showed that the VP1u region of B19V specifically binds and internalizes into erythroid precursor cells, suggesting that the virus uses the RBD in the VP1u for the uptake of the capsid into permissive cell lines [15,16,23]. To verify that the VP1u is capable and sufficient to internalize a capsid structure with the size of B19V into target cells, we sought to crosslink the VP1u to the capsid surface of the bacteriophage MS2 (Figure 1A), thus achieving a retargeting of this unrelated prokaryotic virus to human erythroid cells. 

To this end, we expressed the 130 aa coat protein of MS2 in *E. coli*, which self-assembles into icosahedral capsids with a diameter of about 26 nm [29], and purified the capsids by two consecutive ultracentrifugation steps through 20% sucrose. Since recombinant MS2 proteins semi-specifically encapsidate bacterial RNA, the expressed MS2 capsids can be analyzed by AGE, showing a single nuclease-resistant band at 1.2 kbp (Figure 1B) [30,31]. SDS-PAGE analysis indicated a convenient purity of the assembled coat protein (Figure 1C).

The B19 VP1u was expressed without the C-terminal part (∆C126 VP1u) to exclude the phospholipase A_2_ (PLA_2_) domain and other possibly unknown functional motifs in this region (Figure 1A). Our previous studies have shown that the ∆C126 VP1u has a similar uptake capacity as the wild-type (WT) VP1u protein and thus is sufficient for the internalization [23]. At the C-terminus of the VP1u sequence, we additionally introduced a metal-affinity tag (MAT) for purification, a FLAG tag for immunodetection, and a unique cysteine for site-specific crosslinking.

The coupling of the ∆C126 VP1u with the MS2 capsid was achieved with the heterobifunctional crosslinker NHS-PEG_24_-maleimide. The NHS-group of the crosslinker was coupled to surface lysines on the MS2 capsid, resulting in maleimide-activated MS2. In addition, the capsid was labeled with the fluorophore conjugates NHS-Atto 488 (green) and NHS-Atto 633 (fluorescence in the far red spectrum and visible as blue dye) to follow the capsid in the purification and later in the internalization assays. After removal of the non-coupled crosslinker and fluorophores by size-exclusion, we added the reduced VP1u to allow the reaction of the unique cysteine with the maleimide-activated MS2. SDS-PAGE and Western blot analysis showed that the bioconjugation yielded a crosslinking of at least one VP1u per every 6–8 MS2 protein and about 20–30 VP1u per MS2 capsid, respectively (Figure 1C). Notably, the MS2-coupled ∆N30/∆C126 VP1u partially degraded to a shorter form with a ~∆N70 truncation. Nevertheless, the shorter VP1u still fulfilled the dysfunctional purpose of this construct as a negative control. Furthermore, SDS-PAGE analysis shows that the NHS-PEG_24_-maleimide crosslinker produced a significant number of covalently coupled coat protein dimers, which probably originated from the low reactivity of the maleimide group also for lysine residues on the capsid surface. This intracapsid reaction was particularly favored after the bioconjugation with the VP1u, when the pH of the suspension was raised to pH 8 for hydrolysis and quenching of the maleimide group. AGE analysis showed that the coupling with the PEGylated crosslinker and the VP1u proteins resulted in a slower migration of the capsids, coinciding with the expected larger radius and mass of the bioconjugate (Figure 1B). Furthermore, the Atto 488/Atto 633-labeled capsid proteins (visible as blue bands) migrated with the same retention as the nuclease resistant RNA (visualized with GelRed), showing that the products were labeled MS2 capsids with a uniform size.

### 3.2. The VP1u Region Is Sufficient for Virus Internalization

To test the internalization capacity of the MS2-VP1u construct, we incubated erythroleukemic UT7/Epo cells with the capsid bioconjugate for 1 h at 37 °C and analyzed the samples by fluorescence microscopy and fluorescence-activated cell sorting (FACS). The erythroleukemic UT7/Epo cell line is known to be semi-permissive to B19V infection—the cells allow virus internalization but do not provide a significant production of infectious progeny [4,15,32]. The results showed that the MS2-VP1u construct efficiently internalized into UT7/Epo cells (99.4%), visible as an accumulation of fluorescent signal in the perinuclear region (Figure 1D). The internalization pattern was consistent with the previously reported distribution of B19V or VP1u internalization into the endosomal pathway [15,16,23]. Due to the high fluorescent labeling and the multivalent specificity of MS2-VP1u, the intensity of the signal significantly exceeded the observed signals of the previous studies. Cells with a visible apoptotic morphology did not show uptake of MS2-VP1u (Figure 1D). Importantly, MS2 bioconjugates with a dysfunctional VP1u (∆N30/∆C126) or without an attached VP1u did not internalize, demonstrating that the uptake of capsids was mediated by the B19 VP1u (Figure 1E).

**Figure 2:**
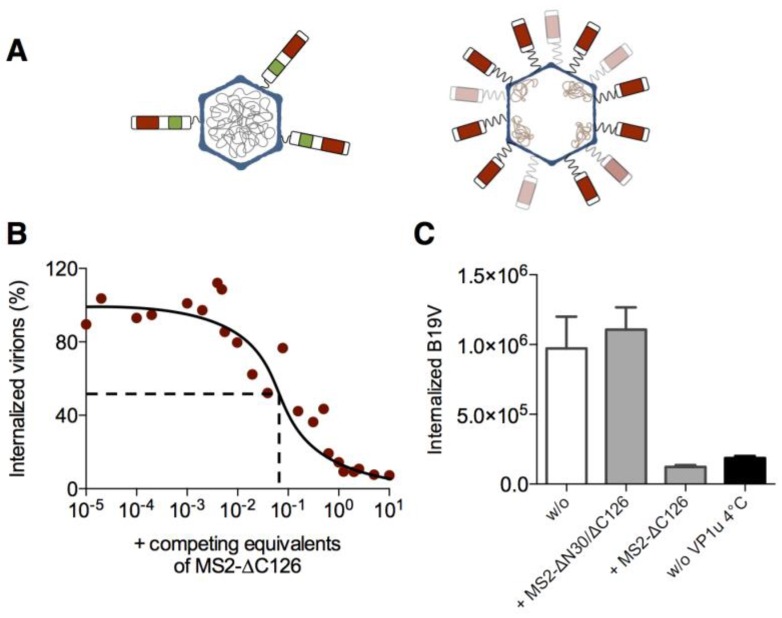
Competition of native B19V internalization with the MS2-VP1u bioconjugate. (**A**) Schematic comparison of the native B19V (three VP1u) with the MS2-∆C126 VP1u bioconjugate (20–30 PEG_24_-linked ∆C126 VP1u per capsid). Nucleic acids are encapsidated in the icosahedral assembled coat proteins (blue). The VP1u contains a phospholipase A_2_ (PLA_2_) motif (green) and a receptor-binding domain (red); (**B**) Internalization of B19V into UT7/Epo cells was measured in presence of increasing concentrations of MS2-VP1u as competitor. After 30 min incubation at 37 °C, cells were washed, trypsinized and internalized B19V DNA was quantified by quantitative polymersaase chain reaction (qPCR; (**C**) Competition of B19V uptake in presence of equimolar concentrations of MS2-∆C126 or internalization-deficient MS2-∆N30/∆C126 (*n* = 3). Samples kept at 4 °C instead of incubation at 37 °C show the background signal without internalization.

To compare the internalization mechanism of MS2-VP1u with the native B19V, we performed uptake competition assays of the two capsids on UT7/Epo cells (Figure 2). To this end, we incubated native B19V with the cells in presence of increasing amounts of MS2-VP1u as an uptake competitor, and allowed endocytosis for 1 h at 37 °C. After repetitive washes and trypsinization—which removes non-internalized particles—the intracellular B19V genomes were quantified by qPCR. The results showed that the MS2-VP1u efficiently competed with B19V uptake (Figure 2B). The concentration required to inhibit B19V internalization by 50% (IC_50_) suggests that the MS2-VP1u was about 10 times more efficient than the native virus uptake. This observation correlates with the number of VP1u per capsid: native B19V capsids contain in average about three VP1u [18]; MS2-VP1u indicated a 20–30 VP1u:capsid ratio (Figure 1C). In comparison, competition of native B19V with VP1u dimers resulted in 50% competition at equimolar concentrations [23]. Importantly, MS2 capsids with the dysfunctional ∆N30/∆C126 VP1u did not compete with native virus uptake, confirming that the uptake competition was governed by VP1u (Figure 2C).

### 3.3. MS2-VP1u Capsids Mimic the Erythroid Specificity of Native B19V

**Figure 3:**
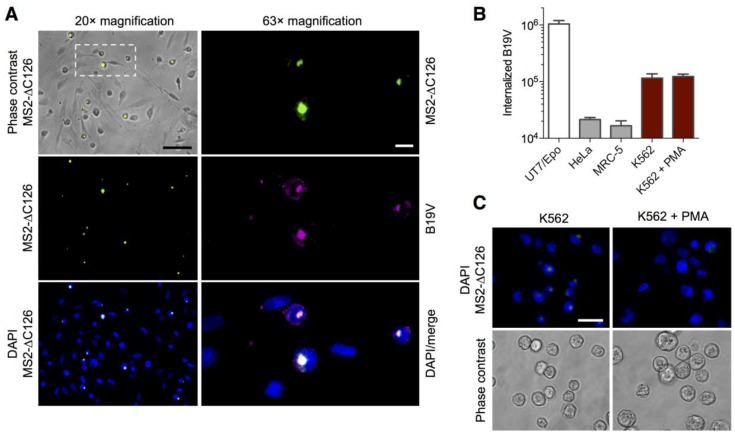
Native B19V and MS2-VP1u specifically target erythroid cell lines. (**A**) MS2-∆C126 VP1u and native B19V were incubated with the same mixed cell culture of non-adherent UT7/Epo cells, and adherent HeLa and MRC-5 cells. Cells were fixed and immunostained with an anti-B19V capsid antibody (860-55D [33]). Cell-specific internalization of MS2-∆C126 VP1u (green) and native B19V (magenta) was visualized by fluorescence microscopy. Scale bar: 100 µm (20× magnification), 15 µm (63× magnification); (**B**) Cellular uptake of native B19V into the different cell lines measured by qPCR (*n* = 3); (**C**) Internalization of MS2-VP1u on normal and phorbol myristate acetate (PMA)-differentiated erythroleukemic K562 cells, visualized by fluorescence microscopy. Scale bar: 30 µm (63× magnification).

To compare the cellular specificity of the MS2-VP1u bioconjugate with the one of the native virus, we analyzed the MS2-VP1u and B19V uptake on different cell lines and mixed cell cultures. The B19V-susceptible, non-adherent erythroleukemic UT7/Epo cells were preincubated with the adherent HeLa cell line and primary MRC-5 lung fibroblasts to obtain a mixed cell population. To avoid competition of B19V with MS2-VP1u, we first incubated the mixed cell culture with B19V for 1 h at 37 °C and then added the fluorescent MS2-VP1u for another hour. After immunostaining of B19V capsids, the cell targeting was visualized by fluorescence microscopy. The results show identical and highly specific cellular internalization of both B19V and MS2-VP1u only into the non-adherent UT7/Epo cells (Figure 3A). In agreement with previous studies [15,16], internalization into the adherent HeLa and MRC-5 cells was not detected. The immunofluorescence result was confirmed by qPCR of B19V internalization into the separate cell lines, showing a clear preference only for the erythroid UT7/Epo cells (Figure 3B). In addition, we also tested the MS2-VP1u and B19V internalization into erythroleukemic K562 cells, which are known to be non-permissive to B19V infection. Similar to our previous results [15,16], these cells supported a very limited uptake of the MS2-VP1u construct (Figure 3C) and further indicated a very low virus internalization signal (Figure 3B), which was comparable to the background levels in UT7/Epo cells (Figure 2). The differentiation of the K562 cells with phorbol myristate acetate (PMA) towards the megakaryblastic lineage – which is associated with the typical giant multinuclear phenotype—resulted in less internalization of the MS2-VP1u and B19V internalization remained on background levels. Taken together, these results suggest that the VP1u receptor is expressed on the erythroid but not on the megakaryocyte lineage.

### 3.4. Expression of the VP1u Receptor Correlates with B19V Uptake and Cellular Permissiveness

**Figure 4:**
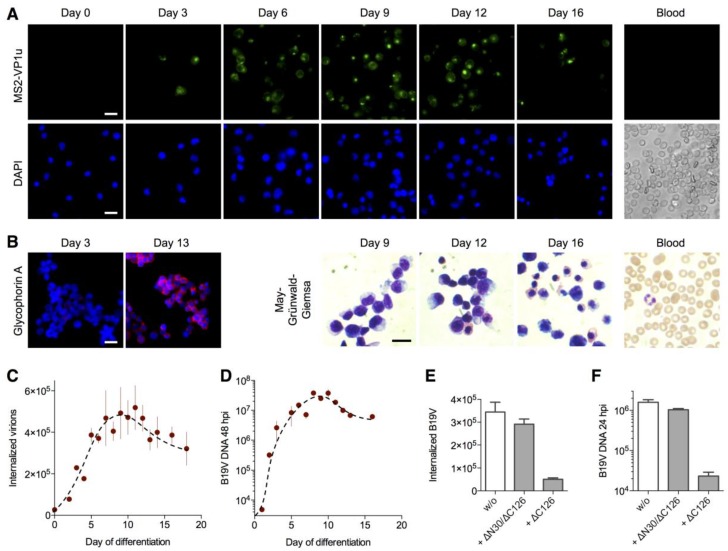
VP1u receptor expression correlates with B19V internalization and infection along the erythroid differentiation: (**A**) cluster of differentiation (CD) 34+ hematopoietic stem cells were expanded ex vivo to differentiate into the erythroid lineage and tested for MS2-∆C126 VP1u uptake (63× magnification). MS2-∆C126 VP1u internalization was further tested on peripheral blood cells, containing mature erythrocytes and ~1% reticulocytes, representing the final erythroid differentiation stages; (**B**) Immunostaining of glycophorin A (63× magnification) and May–Grünwald–Giemsa staining (100× magnification) indicates the successful transition to the terminal erythroid differentiation; B19V internalization (**C**) and infection (**D**) along the erythroid differentiation was quantified by qPCR (*n* = 3); (**E**) Internalization of B19V into erythroid progenitor/precursor cells (day 8) was competed with ∆C126 VP1u or dysfunctional ∆N30/∆C126 VP1u; (**F**) After VP1u-competed uptake of B19V, cells (day 8) were incubated for further 24 h to measure the effect of the competition on the viral infection (*n* = 3). Notably, B19V internalization was performed with more cells (2×) and higher multiplicity of infection (MOI) (7.5×) compared with infectivity assays, explaining the relatively high detection signal of uptake versus infection. Scale bar: 15 µm.

In order to define the correlation of the VP1u receptor expression with the cellular permissivity for B19V infection, we expanded CD34+ hematopoietic stem cells ex vivo towards the erythroid lineage [4,5,6,16] and tested the MS2-VP1u internalization, virus uptake and infection along the differentiation. The results show that MS2-VP1u internalization (Figure 4A), B19V uptake (Figure 4C) and virus infection (Figure 4D) all show a similar profile along the erythroid development. In all cases, the signal increased in the first days from negative to significant amounts, peaking between day 6–11 and then decreasing again to intermediate levels at later stages. Mature erythrocytes and the percentage of reticulocytes in peripheral blood were found negative for the VP1u receptor. To assess the erythroid differentiation during the expansion, we performed a immunostaining with the erythroid marker glycophorin A, as well as a May–Grünwald–Giemsa staining at different days of the development (Figure 4B). Taken together, the finding suggests that the VP1u receptor becomes upregulated in the intermediate erythroid differentiation stages around the proerythroblast stage and strongly correlates with the B19V susceptibility and permissivity.

To evaluate the importance of the VP1u-mediated internalization for viral infection, we competed B19V uptake on day 8 erythroid differentiated cells with an excess of soluble recombinant VP1u and allowed viral replication for 24 h. As expected, the internalization-competent ∆C126 VP1u efficiently interfered with B19V uptake (Figure 4E) and infection (Figure 4F); in contrast, the dysfunctional ∆N30/∆C126 VP1u showed no considerable competition in both assays. This result demonstrates that the VP1u receptor is responsible for the infectious uptake of B19V into permissive cells, and that there exists no significant alternative internalization pathway by another receptor.

### 3.5. The VP1u Receptor Becomes Expressed at the Transition of Burst Forming Unit-Erythroid to the Erythropoietin-Dependent Erythroid Differentiation Stages

**Figure 5:**
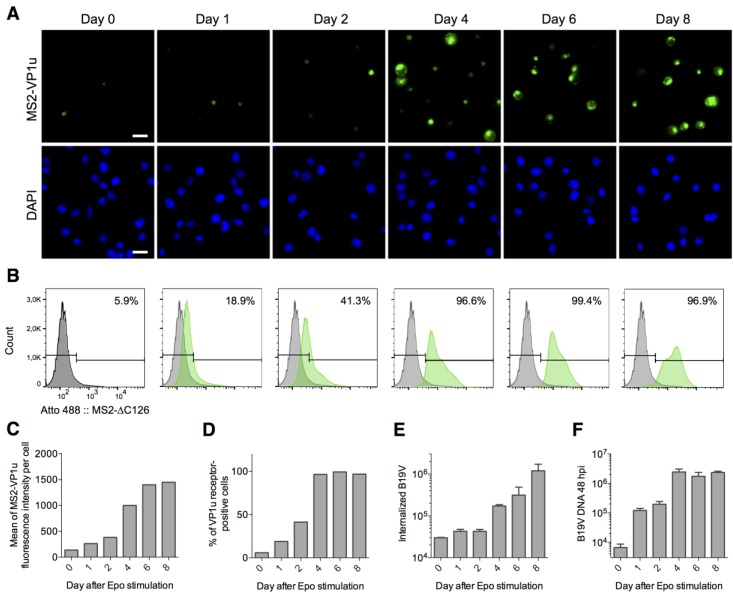
The VP1u receptor is expressed on erythropoietin (EPO)-dependent erythroid differentiation stages: CD34+ hematopoietic stem cells were expanded in StemCell medium and then stimulated with 3 U/mL EPO. VP1u receptor expression, B19V uptake and infection were tested at different days after EPO-stimulation. (**A**) Fluorescence microscopy images and (**B**) flow cytometry analysis of MS2-∆C126 VP1u uptake into differentiated cells; (**C**) Histograms of the FACS data, showing the mean fluorescence of MS2-VP1u internalization per cell and (**D**) number of VP1u receptor-positive cells; (**E**) Quantification of B19V uptake and (**F**) infection before (day 0) or after stimulation with EPO (*n* = 3). Scale bar: 15 µm.

Strikingly, the susceptibility and permissivity of intermediate erythroid differentiation stages overlap with the cellular dependence on the erythroid survival factor erythropoietin (EPO). In line with this, a previous study has shown a strong correlation of EPO signaling, B19V uptake and in particular B19V replication. Therefore, we hypothesized that the VP1u receptor is upregulated in the EPO-dependent differentiation stages, but not in EPO-independent stages as the burst forming unit-erythroid (BFU-E). To this end, we expanded CD34+ cells in StemCell media without EPO for one week to generate a predominant population of the EPO-independent BFU-E stage as previously described [5]. Before and after stimulation with EPO, the cells were analyzed for MS2-VP1u internalization (Figure 5A–D), B19V uptake (Figure 5E), and viral infection (Figure 5F). Besides the visualization with fluorescence microscopy, we quantitatively analyzed VP1u receptor expression by FACS.

As expected, the predominant population of the cells expanded in StemCell media (without EPO) was negative for VP1u receptor expression (94.1% negative, Figure 5B) and further indicated low B19V internalization signal and no significant infection. Upon EPO-stimulation, the total VP1u receptor expression—represented by the mean fluorescence of MS2-VP1u internalization per cell—consistently increased from low levels in the EPO-unstimulated culture to high levels at day 8 after EPO-stimulation. The number of cells that significantly expressed VP1u receptor increased from a subpopulation of 5.9% at day 0 to 96.6% already at day 4. These findings suggest that the VP1u receptor becomes expressed at the transition between the BFU-E and CFU-E stage.

The previous results have demonstrated that VP1u is responsible and sufficient for B19V uptake. Therefore, it was not surprising that the total B19V internalization (Figure 5E) strongly correlated with the level of total VP1u receptor expression along the erythroid differentiation (Figure 5C). Interestingly, the infectivity profile (Figure 5F) rather correlated with the number of VP1u receptor-positive cells (Figure 5D). This result suggests that the expression of the VP1u receptor on a cell—independent of whether its expression is low or high—coincides with the permissivity of the cell.

The expression of intracellular permissivity factors appears to be upregulated at the same moment as the VP1u receptor [5]. The combined expression is exclusively found on the intermediate erythroid differentiation stages whose survival is maintained by the EPO signaling. Without EPO (day 0), only a few differentiated and VP1u-positive cells internalized the virus (Figure 5E); however, without EPO stimulation, these cells were not able to survive and did not support B19V infection (Figure 5F) [5].

Taken together, the VP1u-mediated uptake offers a reasonable and sufficient explanation for the restricted virus internalization into permissive cells and represents a major determinant of the B19V tropism.

## 4. Discussion

B19V shows an extraordinary tropism for erythroid progenitor and precursor cells in the bone marrow. It is thought that this extreme tropism is determined by intracellular permissivity factors as well as by a specific internalization into this cell type [4,5,7]. Previous studies have shown that only erythroid cells around the proerythroblast stage support efficient viral replication and production of infectious progeny [3,4,5,6]. To avoid predominant and abortive infections into non-permissive cells, B19V needs to restrict the virus uptake to permissive erythroid differentiation stages. The expression of globoside as primary receptor generally correlates with the B19V tropism, but cannot explain the selective internalization [5,10]. We have previously reported that the VP1u region mediates the cellular uptake of B19V and that the VP1u receptor appears to be exclusively expressed in the erythroid tissue. Here, we show that the VP1u is sufficient to trigger the highly specific internalization of B19V capsids into EPO-dependent erythroid differentiation stages, and that this cellular uptake consistently correlates with the cellular permissiveness.

In the past, several studies have used ex vivo expansion of CD34+ hematopoietic stem cells isolated from peripheral blood mononuclear cells or bone marrow to define the tropism of B19V along the erythroid differentiation [4,5,6,32,34]. The stimulation of the isolated CD34+ cells with erythropoiesis-supporting cytokines as SCF, IL-3 and EPO generally led to an increase of the permissivity between days 2–4 of the expansion [4,6] (Figure 4D). The observed variation of 1–2 days among the different studies probably derives from the common phenotypic and functional heterogeneity of CD34+ isolated hematopoietic cells depending on the isolation method and source of the primary cells. [35,36]. The studies consistently showed that erythroid differentiation stages around days 7–9 of the expansion provided highest permissivity for B19V infection, coinciding with the CFU-E/proerythroblast stage [2,3,4,5,6]. During the terminal erythroid differentiation stages, permissivity to B19V infection decreased again to intermediate levels.

In this work, we studied the correlation of B19V infection, virus uptake and VP1u receptor expression during erythropoiesis. The results showed that only cells expressing the VP1u receptor also allowed B19V internalization as well as viral infection (Figure 3, Figure 4 and Figure 5). Importantly, the competition assay with recombinant VP1u demonstrated that VP1u-mediated internalization represents the responsible and only significant uptake pathway for B19V infection (Figure 4E,F).

To further define B19V susceptibility and permissivity in the early stages of the erythropoiesis, we used a previously described approach that separates the differentiation state of erythroid progenitors dependent on the cytokine EPO [5]. EPO is known to maintain the survival and proliferation of intermediate erythroid differentiation stages (i.e., CFU-E), proerythroblasts and early basophilic erythroblasts [37,38,39]. The earliest committed erythroid progenitor cells (BFU-E) do not yet show dependence on EPO. BFU-E cells can spontaneously differentiate to the CFU-E stage [40], however, CFU-Es do not survive or proliferate without the EPO signaling and thus remain only as a small subpopulation in StemCell culture [37,39,41]. As a result, ex vivo expansion of CD34+ cells in StemCell media without EPO generates a culture with a predominant population of bipotent megakaryocyte-erythrocyte BFU-E progenitors [5,42,43], which in average only allows a very low B19V entry and basically no infection [5]. Upon EPO signaling, the cells became significantly more susceptible and showed a considerable increase of B19V replication. In line with this, MS2-VP1u only internalized into a small subpopulation of the expanded stem cells (day 0), and upon EPO stimulation an increasing population of cells became VP1u receptor-positive (Figure 5A,B). This result shows that the VP1u receptor is not yet expressed in the BFU-E stage, but becomes upregulated in the EPO-dependent differentiation stages CFU-E, proerythroblasts and early basophilic erythroblasts. The finding further explains why peripheral blood that normally contains a few BFU-E cells [44] was found completely negative for VP1u receptor-expressing cells. Importantly, EPO stimulation does not seem to be the responsible signaling that induces VP1u receptor expression: the small proportion of VP1u receptor-positive CFU-E cells in StemCell media had no contact with EPO, and the EPO-independent K562 cell line shows a very low but significant VP1u receptor expression. It rather appears that EPO signaling allows the survival and proliferation of erythroid cells that just began to express the VP1u receptor.

The expression of the VP1u receptor coincides with the homing of BFU-E cells to erythroblastic islands in the bone marrow and the subsequent differentiation to the immobilized CFU-E stage [43]. This defined chronological expression might indicate a function of the VP1u receptor as an adhesion molecule for the immobilization in the erythroid niche or as a signaling factor for terminal erythroid differentiation steps. Strikingly, the adhesion molecule α5β1 integrin was proposed as a coreceptor for B19V and is known to partially contribute to the adhesion of erythroid precursors in erythroblastic islands [45]. However, the occurrence and properties of the VP1u receptor do not correlate with the expression pattern nor with the reported characteristics of the α5β1 integrin-mediated entry [13]. As an example, α5β1 integrin was reported to support B19V internalization in PMA-treated K562 cells [13]; in our hands, these cells were basically negative for VP1u receptor expression and showed B19V uptake comparable to background signal in erythroid cells. Furthermore, VP1u internalization was not affected by the reported inhibitory antibodies against α5β1 integrin or by the depletion of divalent cations (data not shown). Based on the current state of research it is difficult to suppose α5β1 integrin as the VP1u entry receptor. However, besides the actual tissue expression of cellular receptors, also post-translational modification as glycosylations need to be considered as potential VP1u-binding structures. The identification and physiological function of the VP1u receptor warrants further studies and will not only help to understand the virology of B19V but might also reveal mechanisms involved in erythropoiesis.

The glycosphingolipid globoside was found as a cellular receptor for B19V [8], and shows many characteristics as a primary attachment receptor. The ubiquitous expression of globoside on the erythroid tissue promotes the accumulation of the virus on potentially permissive cells, however, it is not sufficient to trigger virus internalization [5,10,46]. As an example, BFU-E cells generated by ex vivo expansion in StemCell media were reported to express globoside [5] but did not show significant VP1u entry receptor expression (Figure 5A). Hence, early and EPO-independent erythroid progenitor cells might allow virus binding but cannot internalize the capsids (Figure 5E). Besides the preselective accumulation of B19V on target cells, the low affinity binding to globoside might further facilitate tissue spreading and dissemination of the virus by the attachment to circulating red blood cells [8,11,47]. The results suggest a complementary contribution of globoside as a preselective attachment factor and the VP1u receptor as the determinant for the specific virus endocytosis. Together, globoside and the VP1u receptor target and restrict the virus uptake to permissive erythroid cells.

## 5. Conclusions

With the bioconjugation of the VP1u region to the bacteriophage MS2 capsid, we were able to elucidate the role of the VP1u in the internalization process of B19V. We found that the VP1u-mediated internalization determines the erythroid-specific virus uptake. Furthermore, the results showed that the VP1u receptor is simultaneously coexpressed with intracellular permissivity factors in EPO-dependent erythroid differentiation stages. Taken together, the extreme tropism of B19V appears to rely on a restrictive coexpression of cellular receptors and intracellular factors.
